# Urinary metabolomic changes and microbiotic alterations in presenilin1/2 conditional double knockout mice

**DOI:** 10.1186/s12967-021-03032-9

**Published:** 2021-08-16

**Authors:** Jie Gao, Nian Zhou, Yongkang Wu, Mengna Lu, Qixue Wang, Chenyi Xia, Mingmei Zhou, Ying Xu

**Affiliations:** 1 Department of Physiology, School of Basic Medicine, Shanghai University of Traditional Chinese Medicine, 1200 Cailun Road, Shanghai, 201203 China; 2 Center for Chinese Medicine Therapy and Systems Biology, Institute of Interdisciplinary Integrative Medicine Research, Shanghai University of Traditional Chinese Medicine, 1200 Cailun Road, Pudong District, Shanghai, 201203 China; 3 Department of Rehabilitation Medicine, Affiliated Hospital of Nantong University, 20 Xisi Road, Nantong, 226001 Jiangsu China; 4 School of Pharmacy, Shanghai University of Traditional Chinese Medicine, 1200 Cailun Road, Shanghai, 201203 China

**Keywords:** Alzheimer’s disease, PS cDKO mice, Metabolomics, Gut microbiota, 16S rRNA sequencing

## Abstract

**Background:**

Given the clinical low efficient treatment based on mono-brain-target design in Alzheimer’s disease (AD) and an increasing emphasis on microbiome-gut-brain axis which was considered as a crucial pathway to affect the progress of AD along with metabolic changes, integrative metabolomic signatures and microbiotic community profilings were applied on the early age (2-month) and mature age (6-month) of presenilin1/2 conditional double knockout (PS cDKO) mice which exhibit a series of AD-like phenotypes, comparing with gender and age-matched C57BL/6 wild-type (WT) mice to clarify the relationship between microbiota and metabolomic changes during the disease progression of AD.

**Materials and methods:**

Urinary and fecal samples from PS cDKO mice and gender-matched C57BL/6 wild-type (WT) mice both at age of 2 and 6 months were collected. Urinary metabolomic signatures were measured by the gas chromatography-time-of-flight mass spectrometer, as well as 16S rRNA sequence analysis was performed to analyse the microbiota composition at both ages. Furthermore, combining microbiotic functional prediction and Spearman’s correlation coefficient analysis to explore the relationship between differential urinary metabolites and gut microbiota.

**Results:**

In addition to memory impairment, PS cDKO mice displayed metabolic and microbiotic changes at both of early and mature ages. By longitudinal study, xylitol and glycine were reduced at both ages. The disturbed metabolic pathways were involved in glycine, serine and threonine metabolism, glyoxylate and dicarboxylate metabolism, pentose and glucuronate interconversions, starch and sucrose metabolism, and citrate cycle, which were consistent with functional metabolic pathway predicted by the gut microbiome, including energy metabolism, lipid metabolism, glycan biosynthesis and metabolism. Besides reduced richness and evenness in gut microbiome, PS cDKO mice displayed increases in *Lactobacillus*, while decreases in norank*_f_Muribaculaceae*, *Lachnospiraceae_NK4A136_group*, *Mucispirillum,* and *Odoribacter.* Those altered microbiota were exceedingly associated with the levels of differential metabolites.

**Conclusions:**

The urinary metabolomics of AD may be partially mediated by the gut microbiota. The integrated analysis between gut microbes and host metabolism may provide a reference for the pathogenesis of AD.

**Supplementary Information:**

The online version contains supplementary material available at 10.1186/s12967-021-03032-9.

## Introduction

As a disease that mainly affects the elderly, Alzheimer’s disease (AD) accounts for the main part of patients with dementia. About 50 million people were living with dementia in 2018, of which around 35 million were AD patients, and by 2050, 152 million people will be affected by dementia [79]. At present, the incidence of AD in China, which has become the country with the largest number of AD patients globally, has reached more than 8 million [28]. The cost of AD treatment would be a significant financial burden, and the public’s awareness of AD is severely inadequate. Investigating the material basis and the course of the reactions that lead to AD is crucial for understanding the etiology of the disease and developing effective treatment strategies. As the most common neurodegenerative diseases, in addition to neuroinflammation as its principal pathological hallmarks, typical neuropathological features of AD include the accumulation of misfolded amyloid-β (Aβ) peptides, neurofibrillary tangles (NFTs) and tau hyperphosphorylation. However, lots of candidates based on this hypothesis, such as solanezumab, avagacestat, and verubecestat, all failed in the III clinical trials due to the low efficacies, which indicates that the mono-brain-target design is difficult and impossible in the development process of anti-AD drugs. This probably because the theory only presents a partial explanation instead of a holistic understanding of the etiology and pathogenesis of AD.

Recently, accumulating clinical and experimental evidences have suggested that gut microbiota played an essential role in the central nervous system (CNS) diseases through the microbiome-gut-brain axis [56]. It has been reported that multiple pathological features of AD are associated with gut microbiota infections and gut microbiota should be a vital factor for the judgement of the progression of AD [6, 32, 47, 92]. Although it is accepted that the interaction between the gut and brain are bidirectional, the relevant mechanism between the gut microbiota and AD has not yet been fully elucidated. Therefore, it is necessary to conduct in-depth research to clarify the relevant molecular mechanisms.

Changes in the host physiology caused by ageing, genetics, diet, lifestyle and other factors might significantly affect microbes. The 16S rRNA gene sequencing method is a worthy method which could detect fastidiously or non-cultivable organisms by amplifying and confirming the sequence of conserved genes or profiles that not depend on the culture [18, 54]. Metabolomics has emerged as a powerful postgenomic approach in systems biology. It focuses on the study of the small molecule responses of living systems caused by external stimuli from a holistic prospective, providing diagnostic information and mechanistic insight into biochemical effects of drugs. Metabolomics is expected to provide new insights into biomarkers for the diagnosis and prognosis of AD [27, 77, 85]. As a conventional metabolomics research object, urine has also been analysed in metabolomics studies of AD, and characteristic urinary metabolites were identified and considered potential biomarkers of AD [36, 83]. The analysis technology based on gas chromatography-mass spectrometry (GC–MS) with high sensitivity, high resolution and high reproducibility has been widely used in metabolomics studies. The latest studies showed that the host metabolism is affected by the gut microbiota and their metabolites [19, 63]. However, the roles of the gut microbiota and urinary metabolites in AD are still unclear. The combination of 16S rRNA gene sequencing and GC–MS based metabolomics will help to understand the relationship between gut microbiota and CNS diseases from the perspective of the crosstalk of microbiota and metabolites [44, 66, 90].

Presenilin (PS) is a transmembrane aspartate protease. Mutations in PS1gene on chromosome 14 and PS2 gene on chromosome 1 are the principal causes of AD. Cre/LoxP system was used to hybridize the PS1 forebrain specific knockout mice with the PS2 systemic knockout mice to obtain the cDKO mice with double knockout of PS1 and PS2 in the forebrain. The PS 1 and 2 conditional double knockout (PS cDKO) mice began to experience mild cognitive impairment at the age of two months and further showed severe cognitive decline and accompanied by a progressive decrease in learning and memory abilities as a series of AD-like symptoms of neurodegenerative diseases at the age of six months. The PS cDKO mouse model have been utilized for studying the pathological and pharmacological mechanism of AD [16, 55, 67]. Our previous study also has confirmed the benefit of trans-cinnamaldehyde on neuroinflammation and memory deficits in those mice [89]. In this study, we collected the samples of fresh feces and urine from the PS cDKO mice at age of 2 months and 6 months and performed 16S rRNA analyse and GC–MS based non-target metabolomics analysis, respectively.

## Materials and methods

### Experimental animals and sample collection

PS cDKO mice were donated by Dr. Joe Tsien, Augusta University, USA. The generation and genotyping were performed as previously described [60]. 2 and 6 months PS cDKO mice, with the transgene Cre, fPS1/fPS1, and PS2−/−, were served as cDKO-2 and cDKO-6 group (n = 6/group). Their age-matched littermates, without Cre, fPS1/ + and PS2+/+ or fPS1/+ and PS2+/−, were assigned to wild-type controls (WT-2, WT-6) simultaneously. All of the mice were housed in specific pathogen-free environment since born, with ad libitum access to food and water at a temperature of 23 ± 2 °C in 12-h light/dark cycles. In order to avoid the cage effects from microbiome transfer, each group mice were housed individually. Before urine collection, each mouse was individually placed in metabolic cages for 24 h urine.

A total of 32 fecal samples and 32 urine samples were snap-frozen in liquid nitrogen and stored at − 80 °C until analysis. All murine experiments were approved by Animal Experimentation of Shanghai University of Traditional Chinese Medicine and performed in accordance with Animal Care and Use Protocols.

### Urinary metabolomic signatures

The urinary metabolomics study was based on an untarget GC–MS metabolomics method according to our previously published methods with minor modifications [45]. Briefly, 2.0 mL sample was centrifuged at 12,000 rpm for 10 min after thawing the mouse urine sample at room temperature. A 100 μL supernatant was transferred to a 1.5 mL tube, adding 50 μL of urease (148 U/mg, 37 ℃, 15 min) for urea degradation. Then pretreated with myristic acid as an internal standard and N, O-Bis (trimethylsilyl) trifluoroacetamide with 1% trimethylchlorosilane as a derivatizing reagent. The sample analysis was performed using an Agilent 6890/5975B GC/MSD system (Agilent Technologies, Inc. CA, USA).

### Microbiotic community profilings

Total DNA of fecal contents was extracted using E.Z.N.A.®soil DNA Kit (Omega Bio-Tek, Norcross, GA, USA) according to manufacturer’s protocols. Then, the quality and quantity of DNA yield were ascertained by 0.8% agarose gel electrophoresis and the ultraviolet spectrophotometer. By polymerase chain reaction (PCR), the bacterial 16S rRNA gene was amplified with primers 338F (5’-ACTCCTACGGGAGGCAGCAG-3’) and 806R (5’-GGACTACHVGGGTWTCTAAT-3’) targeting V3 to V4 hypervariable region. Products of PCR were evaluated by 2% agarose gel electrophoresis. Residual primers and primer dimers were eliminated with an AxyPrep DNA Gel Extraction Kit (AXYGEN Biosciences, CA, USA) according to the manufacturer’s protocol and quantified using Microplate reader (BioTek, FLx800). Based on the concentration, combine amplicons in an equimolar ratio and performed the paired terminal sequencing on an Illumina MiSeq platform (Illumina, CA, USA) according to standard protocols by Majorbio Biopharm Technology Co. Ltd. (Shanghai, PRC).

### Data analysis

Raw GC–MS raw files (.D) of urine samples were acquired by Chemstation and exported in Net.CDF format. The converted files were subsequently processed by R 2.13.2 (Lucent Technologies). Then the processed data (the peak area, retention time and compounds name) were exported and processed by multivariate analyses including principal component analysis (PCA) and supervised partial least squares discriminant analysis (PLS-DA) using the software of SIMCA-P 11.0 (Umetrics, Umeå, Sweden). Metabolite identification was performed firstly with an already constructed standard library of our lab. Endogenous metabolites from the above-identified compounds were confirmed with online resource HMDB (http://www.hmdb.ca/). The pathways behind identified significant endogenous metabolites were excavated by MetaboAnalyst 3.0 (http://www.metaboanalyst.ca/) [44].

Raw FASTQ files were demultiplexed, qualitifed by fastp version 0.20.0 and merged with the following criteria: (1) regardless of the truncated reads shorter than 50 bp, the 300 bp reads were cut at any site getting an average quality score of < 20 over a 50 bp sliding window; (2) sequences > 10 bp were assembled according to their overlap, and the mismatch ratio of overlap region no more than 0.2; (3) samples were distinguished based on barcode and primers, only accepting 2 nucleotide mismatch in primer matching. Operational taxonomic units (OTUs) with a 97% similarity cutoff were clustered by UPARSE version 7.1 [17] and chimeric sequences were identified and removed. The complete taxonomy of each 16S rRNA gene sequence was analysed by the RDP Classifier version 2.2 [74] with confidence threshold of 0.7. Αlpha diversity was conducted to reveal the diversity indices, including chao1 diversity indices. The β diversity analysis was carried out to explore the similarity or difference of community structure between groups, including principal coordinate analysis (PCoA). The linear discriminant analysis (LDA) effect size (LEfSe) was performed to identify the difference bacterial taxa.

The statistical analyses were performed using two-tailed Student’s t test and Pearson’s multi-variate linear regression analysis by SPSS 25.0. All numerical data are showed as means ± standard deviation (SD). In all experiments, the *p*-value < 0.05 was considered statistically significant.

### Correlation analysis between metabolome and gut taxa

In order to investigate the relationship between gut taxa and perturbed urinary metabolome in PS cDKO mice, correlation coefficient of Spearman’s between metabolome and gut microbiotic alterations in genus level were presented as a heat map. Different color blocks represent distinguish coefficient *r* values. In addition, the metabolic correlation of each relevant member of the gut taxa (|*r*|> 0.4) were expressed in the form of cross-correlation diagram, showing a positive (red line) and negative (blue line) connection.

## Results

### General phenotype of PS cDKO Mice

Numerous previous studies have shown PS cDKO exhibits Alzheimer’s like neurodegenerative symptoms, including cognitive deficits, shrinkage of the cortex, neuroinflammation, and tau hyperphosphorylation in the late stage, despite the decreased Aβ content [8, 60, 81, 89]. These progressions are age related. Although normal in brain morphology, cytoarchitecture, and neuron number, PS cDKO mice at age of 2 months showed mild injury in spatial memory, long-term contextual memory, and synaptic plasticity [60, 78], which may be caused by reduced N-methyl-d-aspartic acid receptor (NMDAR) in synaptoneurosome preparations but not in the entire cerebral cortex [60]. At age of 6 months, they demonstrated a more severe memory impairment and synaptic dysfunction accompanied by the lessened neuronal number and neocortical volume [60, 78, 81]. Besides, mild, but detective loss of gray and white matter along with expansion of the lateral ventricles, hyperphosphorylation of tau and neuroinflammation showed as AD patients [5, 29, 60, 82, 89]. Importantly, these significant early progression of AD in PS cDKO mice at age of 2 and 6 months are very worthy of being explored, considering it is better to treat AD in early progress. Therefore, PS cDKO mice at age of 2 and 6 months were studied to reveal the gut microbiota and urinary metabolome that change with the age.

### Alternation of urinary metabolome in PS cDKO mice

**Fig. 1:**
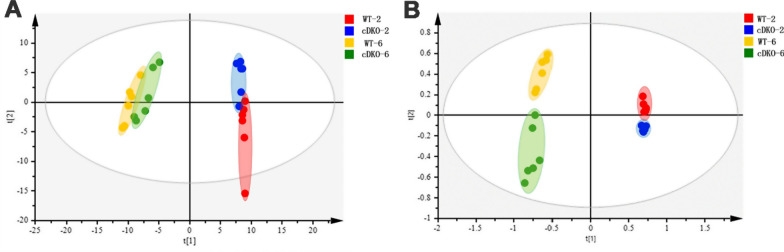
Urinary metabolic profiling (n = 6) of PS cDKO mice and WT mice of 2 months and 6 months. PCA score plot (**A**), PLS-DA (**B**)

**Fig. 2:**
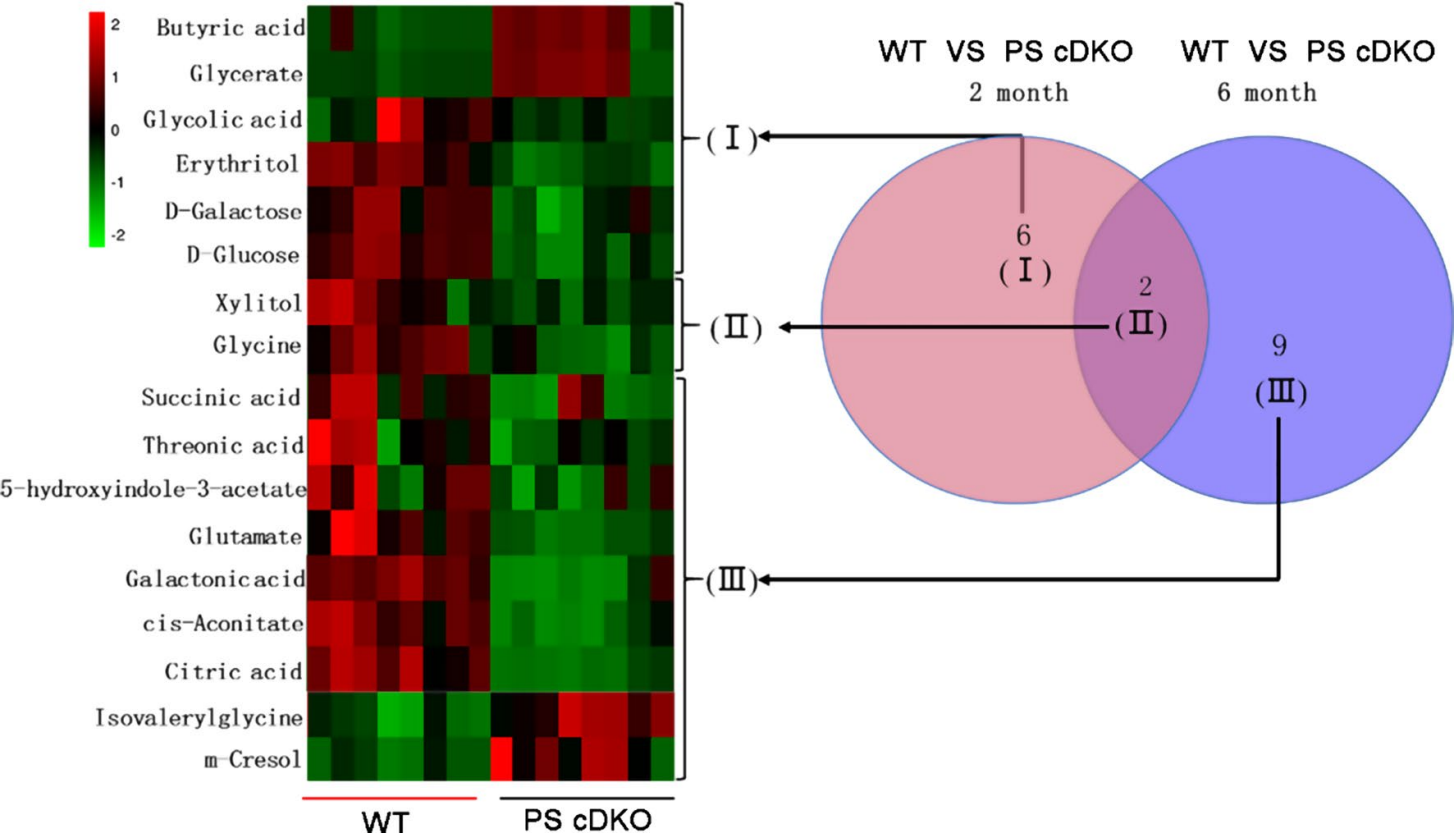
Venn and heat map of the differential metabolites in PS cDKO group and WT group

GC–MS detection based urine metabolomics is an increasingly important approach that has been successfully used to evaluate metabolite changes. The metabolomics results showed clear separations of urine metabolic profiling between PS cDKO mice at the two time points. As shown in Fig. 1, obvious separation was observed between WT and PS cDKO mice in PCA (*R*^2^X = 0.821, *Q*^2^ = 0.508) and PLS-DA (*R*^2^X = 0.864, *R*^2^X = 0.982, *Q*^2^ = 0.959) score plots, suggesting alterations of urinary metabolome in the four groups. In Fig. 2, comparing the group of WT-2 and cDKO-2, a total of 8 differential metabolites were identified. At age of 6 months, the differential metabolites between the WT-6 and cDKO-6 increased to 11. In the above two groups of differential metabolites, 2 of them, Xylitol and Glycine, were coincident and presented as cluster II, which were both downregulated in the two PS cDKO groups. The rest alterations in urinary metabolome of the cDKO-2 group were shown as 6 differential metabolites in Cluster I. Most of them were downregulated, except for the significant upregulation of butyric acid and glycerate. The alterations in urinary metabolome of the cDKO-6 group showed in Cluster II and III. Two out of the 9 differential metabolites in Cluster III were significantly increased, including *m*-cresol, isovalerylglycine. In comparison, the rest 7 metabolites were significantly decreased in the cDKO-6 group, and detailed description of these metabolites was presented in Additional file 1: Table S1. It is worth noting that most of differential metabolites were related to the metabolism of gut microbes, indicating the dysbiosis of gut microbiota in PS cDKO mice.

### Alterations in the metabolic pathway and network analysis

**Fig. 3:**
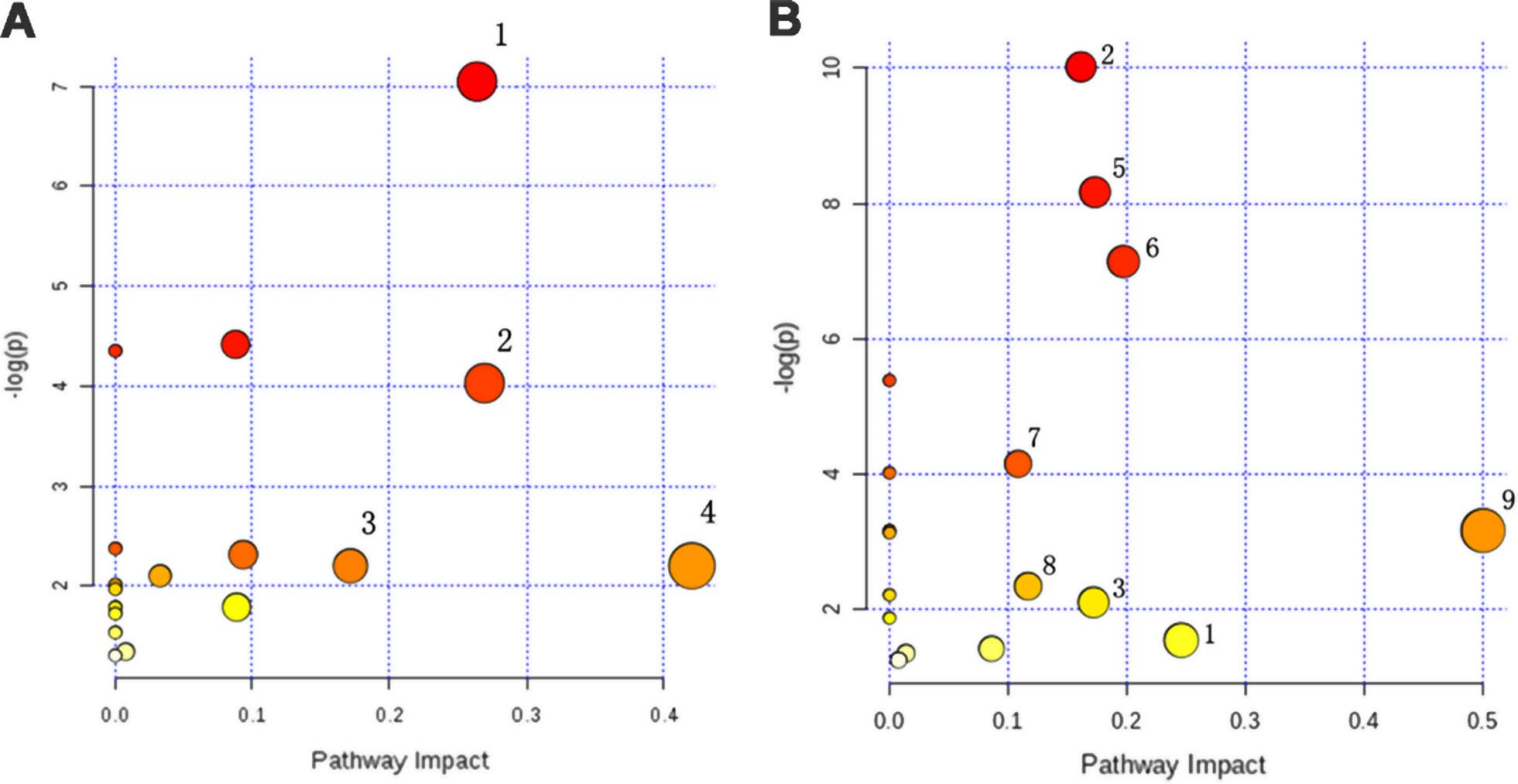
Pathway analysis of urinary differential metabolites using Metaboanalyst (impact factor ≥ 0.1).** A** and **B**: Disturbed metabolic pathways in PS cDKO mice at age of 2 months (**A**) and 6 months (**B**). 1. Glycine, serine and threonine metabolism, 2. glyoxylate and dicarboxylate metabolism, 3. pentose and glucuronate interconversions, 4. starch and sucrose metabolism, 5 citrate cycle (TCA cycle), 6. alanine, aspartate and glutamate metabolism, 7. glutathione metabolism, 8. arginine biosynthesis, 9. d-glutamine and d-glutamate metabolism

**Fig. 4:**
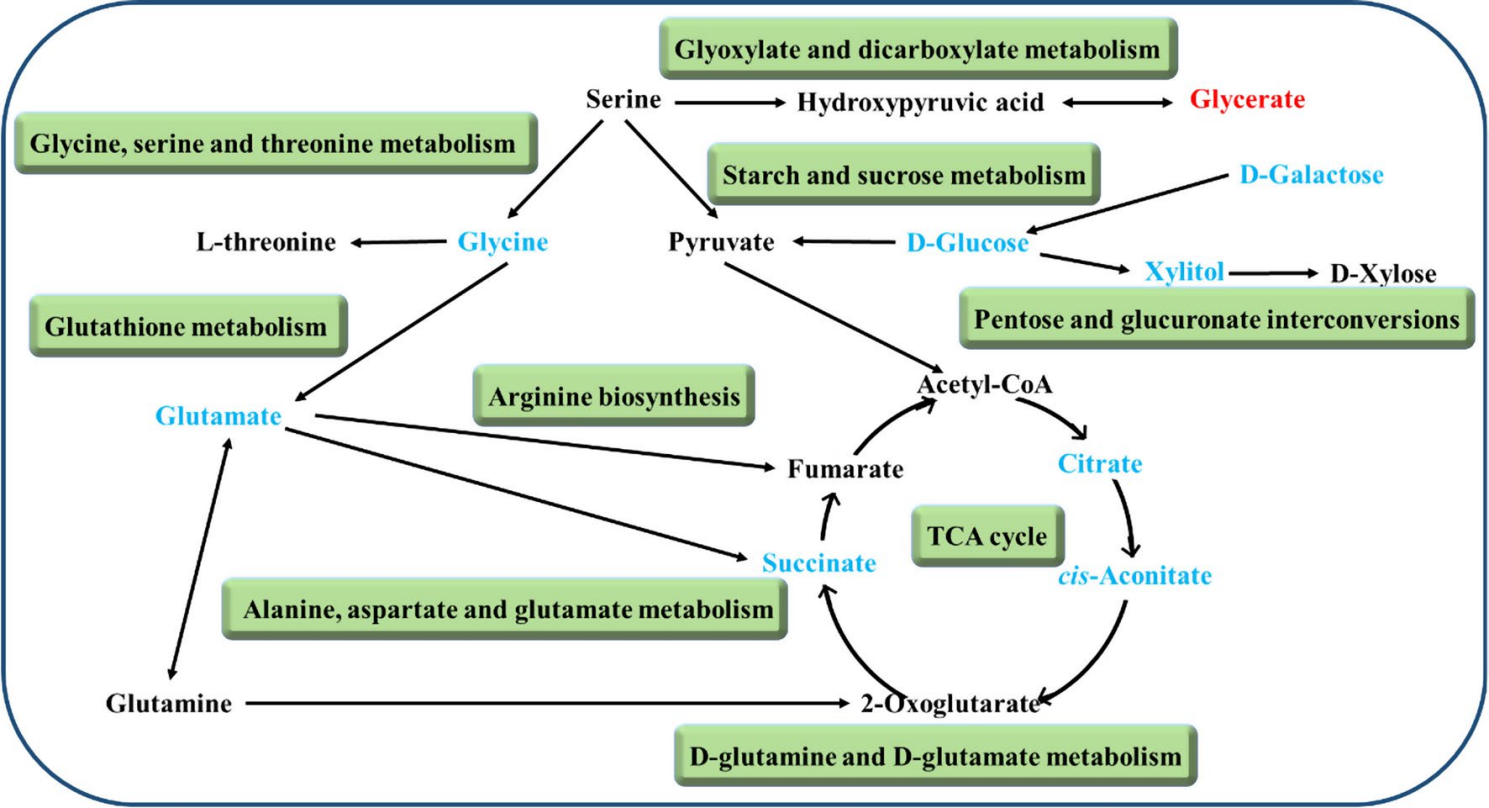
Perturbed metabolic pathways and altered urinary metabolites in PS cDKO mice at age of 2 and 6 months. Red labeled metabolites were up-regulated, and blue labeled metabolites were down-regulated. Green blocks represented disturbed metabolic pathways

The urine differential metabolites were linked to potentially related pathways through KEGG and HMDB database, and their impact values were further identified using MetaboAnalyst 3.0. As a result, four disturbed metabolic pathways were found to be the most relevant pathways involved in 2 months group (impact factor ≥ 0.1). They were glycine, serine and threonine metabolism; glyoxylate and dicarboxylate metabolism; pentose and glucuronate interconversions; starch and sucrose metabolism (Fig. 3A). In addition, we observed that four main metabolic pathways were affected in the 6 months group, they are glycine, serine and threonine metabolism; glyoxylate and dicarboxylate metabolism; pentose and glucuronate interconversions and citrate cycle (TCA cycle) (Fig. 3B). The coincidently disturbed metabolic pathways, glycine, serine and threonine metabolism and glyoxylate and dicarboxylate metabolism, along with glutathione metabolism are related to the significantly lower concentration of glycine in PS cDKO mice at age of 2 and 6 months (Fig. 2). Glycine acts a vital role in the pathogenesis of AD [53, 80], although it is the simplest amino acid. Further, based on the relationship between metabolomic signatures, the disturbed metabolic pathways were represented in diagram (Fig. 4). d-Glucose is the energy substrate necessary to maintain neural activity. Perturbed cerebral glucose metabolism is an eternal pathophysiological feature of AD, and maybe an important factor in its pathogenesis [9]. And d-Galactose, an epimer of d-glucose, may serve as an alternative source of energy. The present study showed that urinary levels of d-glucose and d-galactose were decreased in PS cDKO mice, especially at age of 2 months, which indicates that the early stage of PS cDKO mice is mainly abnormal in brain energy supply. Further, the metabolites, citric acid, cis-aconitate and succinic acid, are substantial components of the TCA cycle. The levels of the above three metabolites in 6-month-old PS cDKO mice are significantly lower than those of the normal mice.

### Alteration of the gut microbiome in PS cDKO mice

**Fig. 5:**
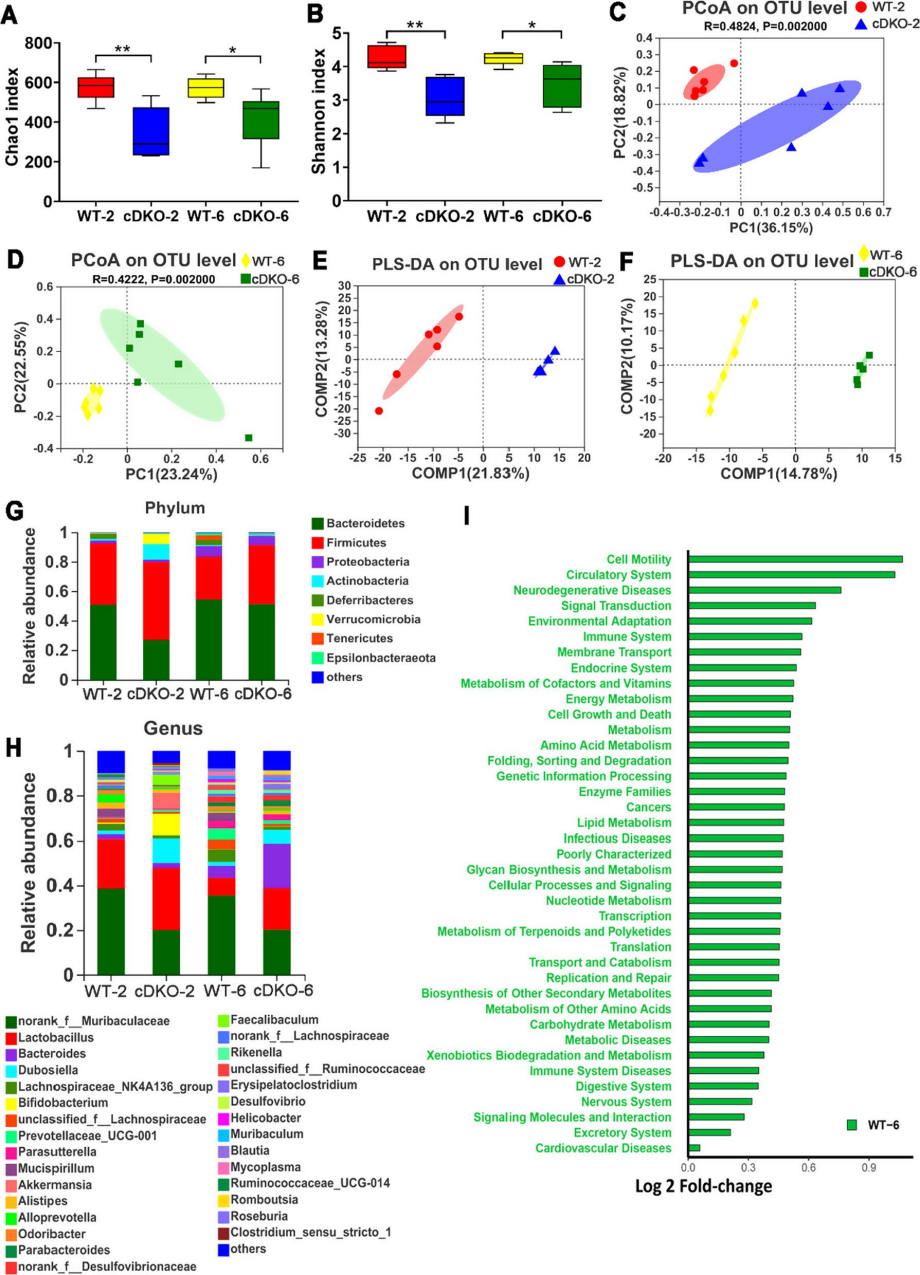
Alteration of gut microbiome in PS cDKO mice. The α-diversity indexes of Chao1 (**A**) and Shannon (**B**) of gut microbiota between PS cDKO and control mice. *, *P* < 0.05, **, *P* < 0.01. PCoA (**C** and **D**) and PLS-DA (**E** and **F**) of the gut microbiome composition of WT and PS cDKO mice on OTU level at age of 2 and 6 months. **G** and **H:** Relative abundance of the gut microbiome in WT and PS cDKO mice at age of 2 and 6 months, coloured at the phylum (**G**) and genus (**H**) level. **I:** The effect of the gut microbiota modifications in PS cDKO mice at age of 6 months compared with age-matched WT mice on predicted functional metabolic pathways acquired from PICRUSt analysis, N = 6

Gut microbiome was accessed by 16S rRNA sequencing (Illumina Miseq platform) using feces samples of cDKO and control groups. The identification of α-diversity index (Chao1 and Shannon) were performed to evaluate the ecological diversity within the microbiotic community. The Chao1 index reflecting community richness were 339 ± 125.84 and 420.44 ± 139.07 in 2- and 6- months old PS cDKO mice, respectively (Fig. 5A). The Shannon index representing both the evenness and species richness was 3.04 ± 0.57 and 3.48 ± 0.63 in 2 months and 6 months aged PS cDKO mice, respectively (Fig. 5B). A significant difference was detected in α-diversity based on Chao1 and Shannon between PS cDKO groups and age-matched WT groups (Fig. 5A, B), which is consistent with the results of the other studies on patients of AD [71]. Moreover, principal coordinates analysis (PcoA) and partial least squares discriminant analysis (PLS-DA) showed the community structure of PS cDKO mice was significantly different from that of WT mice on OTU level (Fig. 5C, D). Early research has reported 5XFAD transgenic mice, a mouse model widely used in AD research, showed that the composition of gut microbiota changed significantly during the progression of AD [75]. In the study of microbiotic community compositions in PS cDKO and WT mice (Fig. 5G, H), *Bacteroidetes* and *Firmicutes* were the two most prominent microbiotic communities at the phylum level, followed by *Proteobacteria*, *Actinobacteria,* and *Daferribacteres* (Fig. 5G). PS cDKO mice seemed to have more *Firmicutes* (an average of 52.80% and 40.07% sequences at age of 2 and 6 months, respectively) and fewer *Bacteroidetes* (26.96% and 51.19%, respectively) than control mice of age-matched, especially in 2-month-old mice. Although at the genus level, the top two microbiome with the highest relative abundance of each group were same. They were norank_f_*Muribaculaceae* (the microbiome, norank in the genus, but belonging to the *Muribaculaceae* family) and *Lactobacillus* (Fig. 5H). The community structures were different in each group. Compared with age-matched control groups, the abundance of norank_f_*Muribaculaceae* was significantly reduced in the cDKO groups at 2 and 6 months. Both PS cDKO and WT mice showed that *Lactobacillus* decreased with age, but the difference in abundance was more pronounced at 6 months of age (Fig. 5F). In general, bacterial abundance and community structure between PS cDKO and WT mice were significantly various, and the bacterial community structure became complicated with age. PICRUSt analysis showed that the differences in gut microbiota between WT-6 and cDKO-6 affected the prediction of functional metabolic pathways (Fig. 5I). Compared with age-matched PS cDKO mice, the gut microbiota of WT mice at 6 months was involved in 39 higher metabolic pathways, including signal transduction, energy metabolism, lipid metabolism, glycan biosynthesis and metabolism, and cellular processes and signaling, which were previously observed in the impairments of AD mice model or associated with the disease progression of AD [1, 35, 38, 89].

### Alterations of the gut taxa in PS cDKO mice

**Fig. 6:**
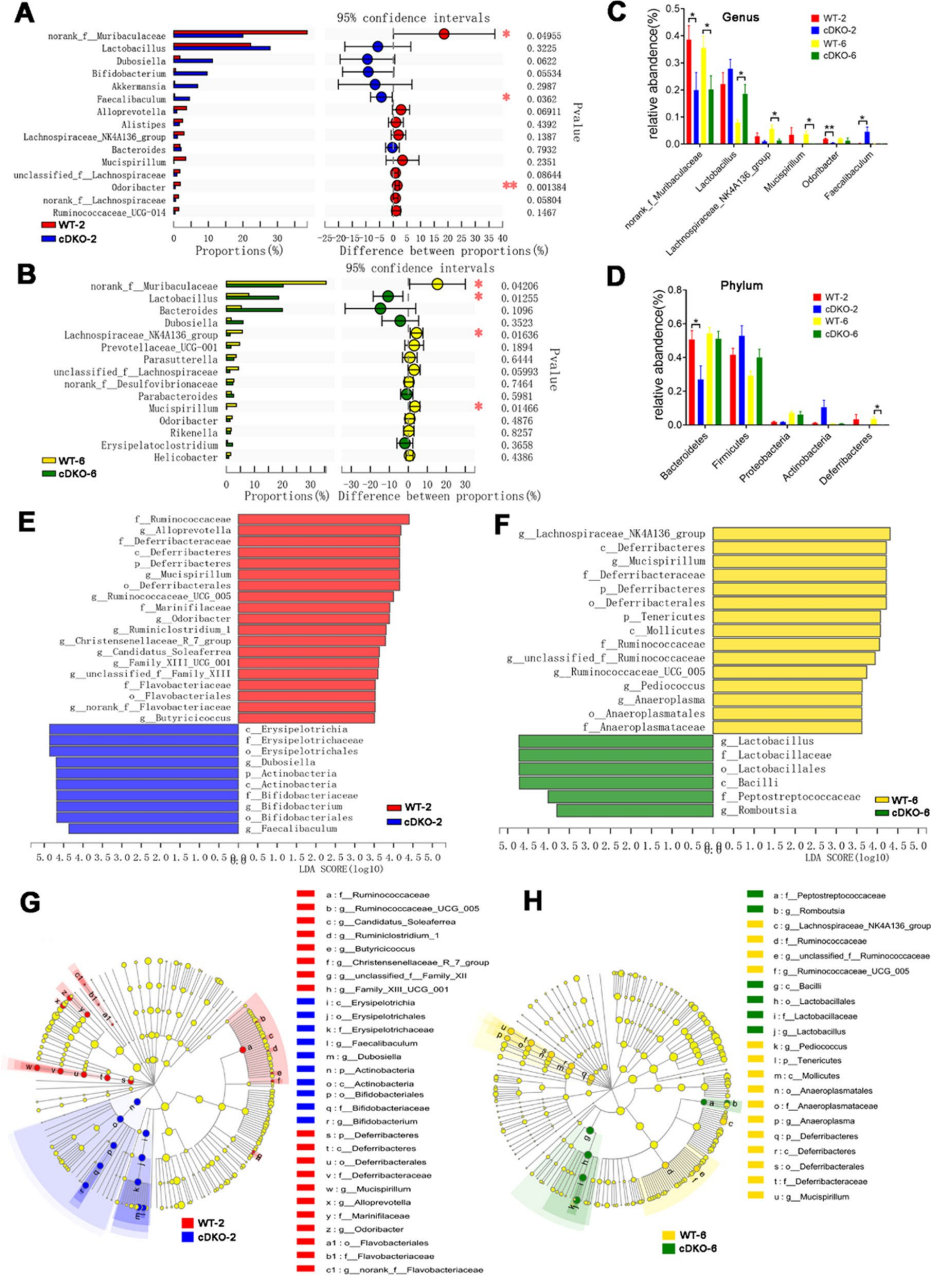
The differentially taxa of gut microbiota in PS cDKO mice versus WT mice. Differential abundance analysis of taxa on the genus level (top 15) between WT and PS cDKO mice at age of 2 months (**A**) and 6 months (**B**). Student’s t-test, ∗ *P* < 0.05, ∗  ∗ *P* < 0.01. **C:** Comparison of relative abundance of significantly altered bacterial taxa on genus level between WT and PS cDKO mice. ∗ *P* < 0.05, ∗  ∗ *P* < 0.01. **D:** Comparison of relative abundance of gut microbiota on the genus level (top 5) between WT and PS cDKO mice. ∗ *P* < 0.05. LEfSe analysis from the phylum to genus level at age of 2 months (**E**) and 6 months (**F**). Taxa enriched in PS cDKO mice are indicated by a negative LDA score (blue for cDKO-2 group; green for cDKO-6 group), and taxa enriched in WT groups have a positive LDA score (red for WT-2 group; yellow for WT-6 group). The LDA score threshold is ≥ 3.5. **G** and **H:** The cladogram of enriched taxa from the phylum to genus level, N = 6

In terms of the community structure and the taxonomy level, the differences between PS cDKO and WT mice were not significant. Therefore, the top 15 gut microbiomes of them at genus level were compared respectively. LEfSe was used to identify the differential bacterial taxa from phylum to genus. At the genus level, the relative abundance of norank*_f_Muribaculaceae*, *Faecalibaculum* and *Odoribacter* were significantly different between the cDKO-2 and WT-2 groups (Fig. 6A). However, the difference in the gut microbiota between the 6-month-old PS cDKO and WT mice had changed. In addition to the difference in norank*_f_Muribaculaceae*, the relative abundance of *Faecalibaculum* and *Odoribacter* no longer had a significant difference (Fig. 6B). In contrast, the relative abundances of *Lactobacillus*, *Lachnospiraceae_NK4A136_group* and *Mucispirillum* were significantly different (Fig. 6A, B). Further, the differential gut microbiota with age changes between PS cDKO and WT mice was observed. Both at age of 2 and 6 months, PS cDKO mice had lower levels of norank*_f_Muribaculaceae* (Fig. 6C), primitively called S24-7, belonging to phylum *Bacteroidetes*. Besides, as described above, less *Lactobacillus* and a higher rate of *Lachnospiraceae_NK4A136_group* and *Mucispirillum* were observed in 6-month-old PS cDKO mice, but the difference was not significant at age of 2 months. Interestingly, compared with age-matched WT mice, the enrichment of *Odoribacter* was reduced in 2-month old PS cDKO mice and the incidence of *Faecalibaculum* was also increased. However, in 6-month-old mice, there was no significant difference.

LEfSe analysis showed that the 2-month old PS cDKO mice were primarily characterized by higher abundance of *o_Erysipelotrichales*, *c_Erysipelotrichia*, *f_Erysipelotrichaceae*, *g_Dubosiella*, *c_Actinobacteria*, *p_ Actinobacteria*, *g_Bifidobacterium*, *o_Bifidobabacterials*, *f_Bifidibacteriaceae* and *g_Faecalibaculum* (LDA score ≥ 3.5) (Fig. 6E), whereas WT mice mainly showed higher enrichment of 19 other microbiota from phylum to genus. Surprisingly, there were only 6 taxa with LDA score ≥ 3.5 in 6-month-old PS cDKO mice, namely *g_Lactobacillus*, *f_Lactobacillaceae*, *o_Lactobacillales*, *c_Bacilli*, *F_Peptostreptococcaceae,* and *Romboutsia*, while there were 15 other taxa with LDA score ≥ 3.5 in WT-6 group (Fig. 6F). Cladogram clearly demonstrated the hierarchical relationship among the main gut bacteria taxa in PS cDKO and WT mice (Fig. 6G, H). For example, in WT mice, taxa from *p_Deferribacteres* to *g_Mucispirillum* were all abundant (Fig. 6G, H). With the increment of age, the trend of changes in *Deferribacteres* and *Mucispirillum* was similar (Fig. 6C, D). In addition, compared with age-matched WT mice, 6-month-old PS cDKO mice were rich in *Lactobacillus*, which belongs to *Lactobacillaceae* at the family level, and their order-level of *Lactobacillales* and class-level of *Bacilli’s richness* were higher (Fig. 6F).

### Correlation analysis of metabolomic signatures and microbiotic community profilings

**Fig. 7:**
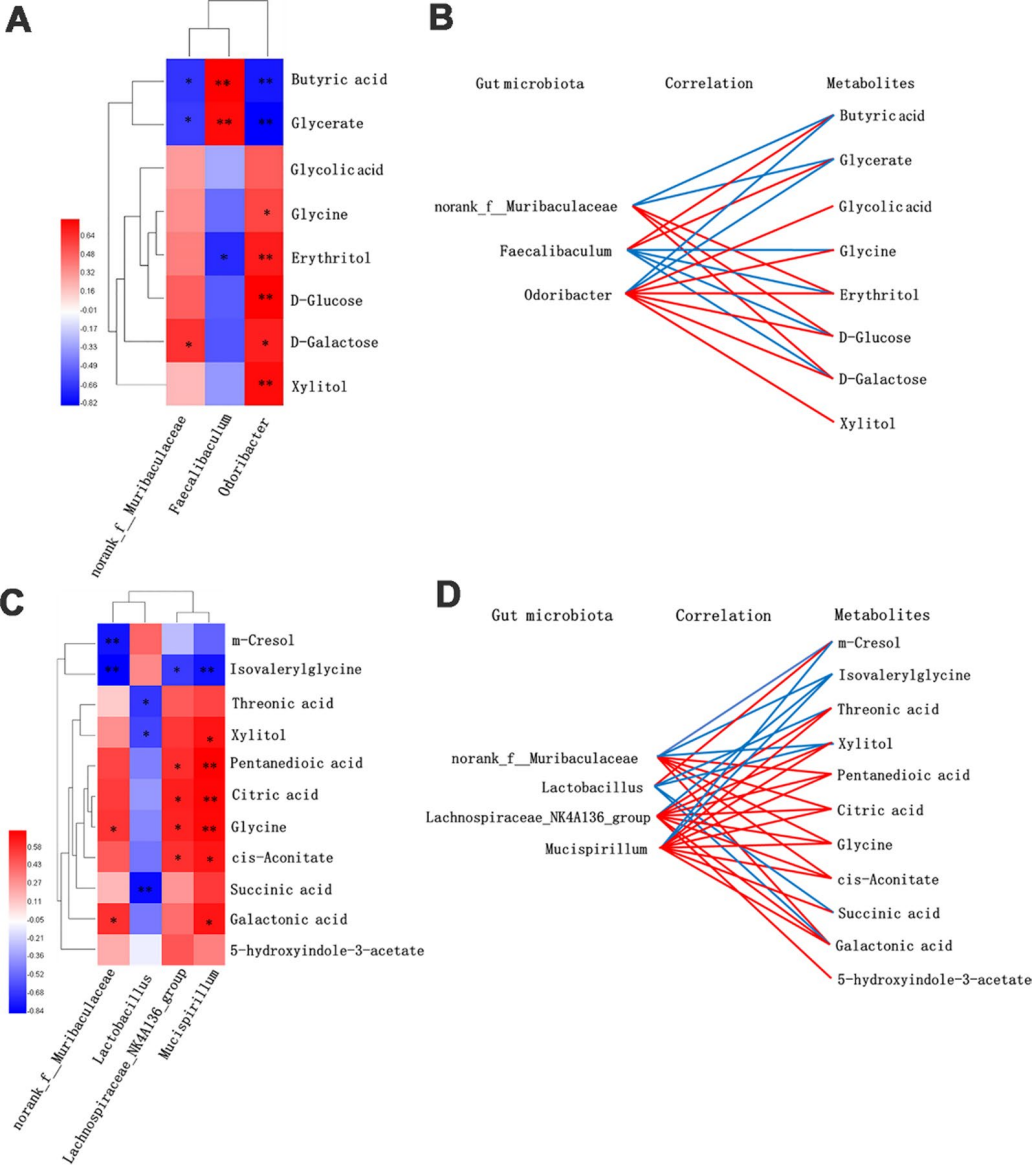
The relevance between the gut microbiota of genus level and the differential urinary metabolites.** A** and **C:** Spearman’s correlation heat map: red indicates positive correlation, while blue indicates negative correlation. The deeper color means the greater correlation (* *P* < 0.05, ** *P* < 0.01). **B** and **D:** The gut microbiota of genus level, predicted by metabolic variation (|*r*|> 0.4), is labeled with a similarity value. Lines connecting with metabolites show the direction of relevance to each genus of microbe with the red (positive) or blue (negative) lines

In order to further understand the correlation of metabolomic characteristics and intestinal microbiotic communities, the covariation relationships between urinary differential metabolites and the differential gut microbiota of genus level were expressed in the form of heat diagram (Fig. 7A, C). The correlations between differential gut microbiota of genus level and differential metabolites were multiple, and the metabolic connections of well-predicted bacteria were shown in Fig. 7B, D (|*r*|> 0.4). In 2-month-old groups of WT and PS cDKO, norank_f_*Muribaculaceae* was negatively correlated with butyric acid (*r* =  − 0.650, *P* < 0.05) and glycerate (*r* =  − 0.629, *P* < 0.05), and positively correlated with erythritol (*r* = 0.406), d-galactose (*r* = 0.650, *P* < 0.05), and d-glucose (*r* = 0.503). *Faecalibaculum* has a positive correlation with butyric acid (*r* = 0.804, *P* < 0.01) and glycerate (*r* = 0.776, *P* < 0.01), but negatively related to glycine (*r* =  − 0.476), erythritol (r =  − 0.685, P < 0.01), d-galactose (*r* =  − 0.545), and d-glucose (*r* =  − 0.524). *Odoribacter* was negatively correlated with butyric acid (*r* =  − 0.741, *P* < 0.01) and glycerate (*r* =  − 0.818, *P* < 0.01), but positively correlated with glycolic acid (*r* = 0.510), xylitol (*r* = 0.769, *P* < 0.01), glycine (*r* = 0.587, *P* < 0.05), erythritol (*r* = 0.720, *P* < 0.01), d-galactose (*r* = 0.706, *P* < 0.05), and d-glucose (*r* = 0.804, *P* < 0.01). In 6-month-aged group, norank*_f_Muribaculaceae* was negatively correlated with m-cresol (*r* =  − 0.776, *P* < 0.01) and isovalerylglycine (*r* =  − 0.839, *P* < 0.01), and positively correlated with glutamate (*r* = 0.531), cis-aconitate (*r* = 0.462), citric acid (*r* = 0.559), galactonicacid (*r* = 0.594, *P* < 0.05), and glycine (*r* = 0.580, *P* < 0.05). *Lactobacillus* and m-cresol was positively correlated, and threonic acid (*r* =  − 0.671, *P* < 0.05), glutamate (*r* =  − 0.441), xylitol (*r* =  − 0.629, *P* < 0.05), cis-aconitate (*r* =  − 0.476), galactonic acid (*r* =  − 0.462), glycine (*r* =  − 0.420), and succinic acid (*r* =  − 0.804, *P* < 0.01) were negatively correlated. *Lachnospiraceae*_NK4A136_group was negatively correlated with isovalerylglycine (*r* =  − 0.657, *P* < 0.05), but positively correlated with threonic acid (*r* =  − 0.657, *P* < 0.05), glutamate (*r* =  − 0.657, *P* < 0.05), 5-hydroxyindole-3-acetate (*r* =  − 0.657, *P* < 0.05), xylitol (*r* =  − 0.657, *P* < 0.05), cis-aconitate (*r* =  − 0.657, *P* < 0.05), citric acid (*r* =  − 0.657, *P* < 0.05), galactonic acid (*r* =  − 0.657, *P* < 0.05), and glycine (*r* =  − 0.657, *P* < 0.05). *Mucispirillum* was negatively correlated with m-cresol (*r* =  − 0.531) and isovalerylglycine (*r* =  − 0.776, *P* < 0.01), and positively correlated with threonic acid (*r* = 0.531), glutamate (*r* = 0.741, *P* < 0.01), xylitol (*r* =  − 0.657, *P* < 0.05) cis-aconitate (*r* = 0.678, *P* < 0.05), citric acid (*r* = 0.727, *P* < 0.01), galactonic acid (*r* = 0.678, *P* < 0.05), glycine(*r* = 0.720, *P* < 0.01), and succinic acid (*r* = 0.566).

**Fig. 8:**
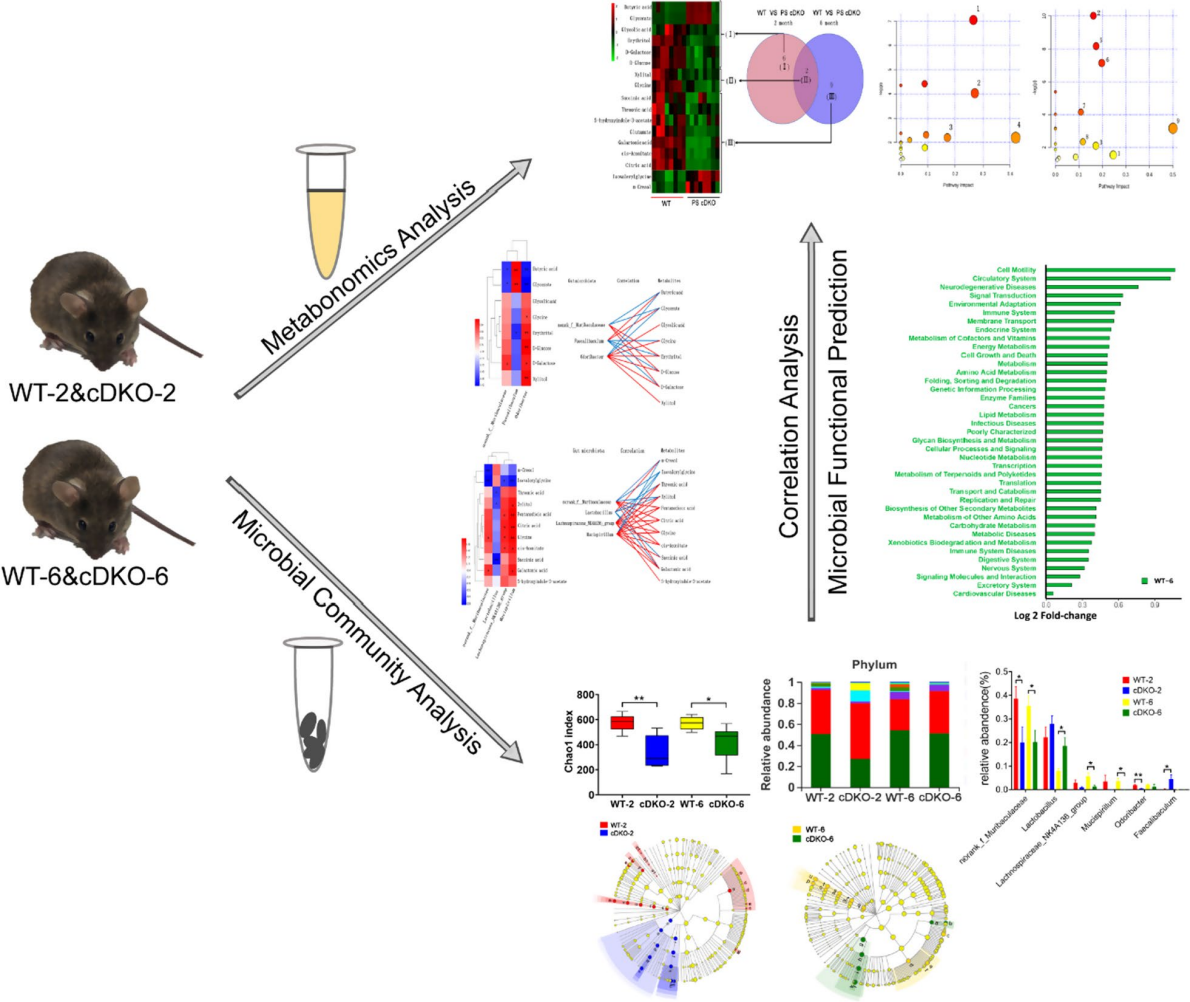
Schematic diagram representing metabolomic and microbiotic changes of PS cDKO mice and the effects of gut microbiota on mediation of urinary metabolomic changes

## Discussion

Emerging evidences have proved that gut microbiota and the brain show bidirectional interaction through microbiota-gut-brain axis [12, 91]. The presenilins are essential components of the multiprotein γ-secretase complex, which regulates embryonic neurogenesis [15]. The aberrant presenilin genes are associated with familial-Alzheimer’s disease, as well as increased inflammatory responses and age-related neuropsychiatric symptoms, such as anxiety, depression, aggressivity, and aberrant motor behavior [13, 81]. Due to deficient β-catenin phosphorylation and notch signaling, the knockdown of PS1 in neural progenitor cells (NPCs) are causally linked to cognitive impairment [4], and the additional loss of PS2 will exacerbate aberrant premature differentiation of NPCs [15]. Although the essential role of presenilin genes or gut microbiota in the pathogenesis of AD has been widely reported, so far, there are few reports on the structural characteristics of gut microbiota in organisms with aberrant PS genes and their relationships with the host metabolic phenotype. In the present study, integrative metabolomic signatures and microbiotic community profilings were applied on presenilin1/2 conditional double knockout (PS cDKO) mice of its early age (2-month) and mature age (6-month), comparing with gender and age-matched C57BL/6 wild-type (WT) mice.

Amino acid neurotransmitters, usually divided into excitatory and inhibitory types, are crucial in neurotransmission, and closely related to the organisms’ learning and memory ability. Recently, some studies have reported that aberrances in amino acid metabolism can be an early indicator of neurodegeneration in AD [20, 30, 76]. Glycine and glutamate are agonists of N-methyl-d-aspartate (NMDA) receptors [21, 25], the activation of which is essential for triggering memory [48]. It was reported that glycine transporter-1 (GlyT1) inhibitors could improve glycine uptake and moderate cognitive impairment in animal models of Alzheimer's disease [21]. Our previous study found that PS cDKO mice have fewer NMDA receptors in prefrontal cortex and hippocampus [89]. In this study, it was also demonstrated that the urine levels of glycine and glutamate were reduced (Fig. 2). Besides, as an important excitatory neurotransmitter, glutamic acid can be used to inhibit the energy formation and biosynthesis of the inhibitory mediator GABA [52]. Previous animal and clinical studies reported AD model animals or AD patients had a lower level of glutamate in the brain [2, 57]. In patients with mild cognitive impairment (MCI), it was also reported that glutamate levels decreased over approximately 12 months in the posterior cingulate gyrus [50].

Differential metabolites identified in the metabolomics studies were mainly related to disorders of energy metabolism. And importantly, energy metabolism plays a significant role in AD. Accumulated evidence indicated that both oral and subcutaneous administration of d-galactose had positive effects on learning and memory abilities of Wistar rats and streptozotocin-induced AD models [11, 33, 58]. Citric acid, *cis*-aconitate and succinic acid are vital compounds in the physiological process of converting fat, protein and sugars into carbon dioxide. These chemical reactions are the core of almost all metabolism organisms and provide energy for them [9, 43]. These reduced three metabolites of PS cDKO mice further suggested that energy metabolism is a crucial factor in AD.

As a naturally occurring sugar acid, L-threonate (threonic acid) is generally excreted in urine by about 10% [69]. Threonic acid could affect the CNS. For example, synapse density and memory ability could be improved in the elderly rats and APPswe/PS1dE9 (APP/PS1) transgenic mice by oral administration of L-threonate and magnesium (Mg ^2+^) in the form of L-threonine magnesium salt (L-TAMS) [39, 64]. In addition, the elderly with cognitive impairment can also get improved cognitive ability by oral intake of a compound containing L-TAMS [42]. These can partly explain the decrease of threonic acid levels in the 6-month-old PS cDKO group (Fig. 2). Based on the above analysis, it can be concluded that there are significant differences in the metabolites of AD at different stages. This indicates that in the future, specific biomarkers developed from certain differential urinary metabolites can be used to distinguish the different disease courses of AD. Of course, further confirmatory research needs to be carried out extensively before that.

AD is accompanied by alterations in the gut microbiotic community [75, 92], which was further confirmed in our present study. Significant genus-level changes included increases in *Lactobacillus*, while decreases in norank*_f_Muribaculaceae*, *Lachnospiraceae_NK4A136_group*, *Mucispirillum,* and *Odoribacter. norank_f_Muribaculaceae*, belonging to phylum *Bacteroidetes*, is positively related to the formation of the inner mucus layer and its barrier function in the gut [72]. Meanwhile its abundance was closely correlated with propionate, a kind of short-chain fatty acids (SCFAs) in feces [65]. In our study, the abundance of the genus *Lachnospiraceae_NK4A136_group* in PS cDKO mice was decreased, which is also a SCFAs-producing bacteria [87, 88]. SCFAs not only perform on the gut, but also act on the brain and other distal places, modulating permeability of the blood–brain barrier, neurogenesis, and host behaviors [34]. SCFAs activate intestinal gluconeogenesis via a cAMP-dependent mechanism or through a gut-brain neural circuit involving the free fatty acid receptor (FFAR3) [14]. It was reported that the level of SCFAs in feces and brain of AD mice was reduced [86], and selective SCFAs could interfere with the formation of toxic soluble Aβ aggregates in vitro [24]. Besides, the decreased abundance of *Lachnospiraceae_NK4A136_group* was also observed in hydrocortisone-induced depressant rats [10]. *Lactobacillus*, a pivotal component of the *Firmicutes* phylum, usually affects the immune system [70]. *Lactobacillus plantarum TW1-1*, isolated from a fermented milk product, was reported to modulate gut microbiota and have anti-inflammatory and anti-oxidative stress activities [70]. The abundance of *Lactobacillus in* PS cDKO mice was higher than in WT mice, especially in 6-month-old PS cDKO mice (Fig. 6). It has also been same reported that in P301L mice, an AD model with tauopathy, *Lactobacillus* was increased [68].

*Odoribacter* belongs to *Bacteroidetes* phylum, and its variation between PS cDKO and WT mice was the same as that of *Bacteroidetes*. One of the novel metabolite markers of *Odoribacter*, sulfonolipids has broad application prospects in biological fields, such as intestinal maturation and immune system [73]. Sulfobacin *B*, a kind of sulfonolipids, could inhibit inflammatory responses by decreasing the production of TNF-α [46]. In our study, the level of *Odoribacter* was lower in both 2-month-old and 6-month-old PS cDKO mice, which are consistent with the downward trend of the immune system of PS cDKO mice. As the sole known component of *Deferribacteres in* the mammalian microbiota, the abundance of *Mucispirillum* was reduced, as shown in LEfSe analysis (Fig. 6). *Mucispirillum* is related to the pathogen invasion and virulence factor expression partially by competing for anaerobic electron acceptors [23] and plays a causal role in protection against *Salmonella*-induced colitis. In our study, the abundance of *Mucispirillum* was decreased in the PS cDKO mice, which might be associated with the complicated chronic inflammation in AD. The enrichment of *Deferribacteres* is closely related to the protection against *Salmonella enterica* serovar Typhimurium (*S*. Tm) colitis [23]. At the same time, the abundance *of Deferribacteres* was decreased in mice with constipation predominant intestine tumor, and could be reverted after treatment [40]. It could gain energy by obligatory or facultative anaerobic metabolism [40]. Therefore, *Deferribacteres* is a kind of beneficial bacteria, and the decrease of phylum *Deferribacteres* in the gut may be related to the disease progression of AD.

With the integration of gut microbiota data with differential metabolites, the tight crosstalks between the gut microbiota and host metabolism in the PS cDKO mice of different ages were highlighted. *Faecalibaculum rodentium*, a typical species of *Faecalibaculum* and a Gram-positive and obligately anaerobic bacteria, has a higher fermentation ability for lactic acid, as well as butyric acid production, and is hypothesized to be the principal replacement of *Lactobacillus* and *Bifidobacterium*, along with a shift from lactate metabolism to increased SCFA production and carbohydrate metabolism [7, 41]. Endogenous murine *Faecalibaculum rodentium* was proved to protect from intestinal tumor growth by producing anti-proliferative SCFAs, especially that butyrate [84]. *Odoribacter*, Gram-negative and strictly anaerobic, contains only two recognized species, namely *O. splanchnicus* and *Odoribacterdenticanis* [22]. Its metabolites include not only butyric acid, but also small amounts of succinic acid, acetic acid, and isovalerate acid [49]. As a beneficial bacteria in the gut, *Odoribacter* can protect the intestinal immune system from external stimulation and enhance the intestinal mucosa’s function [87, 88]. In our present study, the results show that *Faecalibaculum* and *Odoribacter* were relevant to the differential metabolites of PS cDKO mice, which represented the disturbances of metabolic pathways that accompanied the disease development of AD. Norank*_f_Muribaculaceae*, a longevity-linked bacterial family [61, 62], contains functional genes that are related to the degradation of particular types of polysaccharides, such as a-glucans, host glycans and c plant glycans [37, 51], and the production of succinate, acetate, and propionate [51]. In this study, the decreased abundance of *Muribaculaceae* was consistent with the decreased levels of energy and lipid metabolism in PS cDKO mice in the metabolomics study and the related clinical manifestations of AD patients [3, 31].

*Lactobacillus* metabolize sugars into lactic acid, which lowers the pH of their environment. Generally, it has an anti-bacterial and anti-fungal function and is regarded as a probiotic. It was reported that the percentage of *Lactobacillus* spp. within the general gut microtiota was higher in Tsumura Suzuki Obese Diabetes (TSOD) mice than in Tsumura, Suzuki, Non Obesity (TSNO) mice [26]. In contrast, the percentages of family *Lachnospiraceae* were higher in TSNO mice than in TSOD mice [26]. At age of 6 months, Lactobacillus was higher in PS cDKO mice (Fig. 6C), which may be related to the abnormal glucose metabolism in PS cDKO mice and the decrease of the OTU richness (Chao1) and diversity index (Shannon). Figure 7C showed that *Lactobacillus* was negatively relevant to threonic acid, xylitol and succinic acid, revealing the TCA circle that decreases with age. *Lachnospiraceae_NK4A136_group* and *Mucispirillum* both were negatively related to isovalerylglycine, and positively related to glutamate, citric acid, glycine and *cis*-aconitate. The decrease of these two bacteria was related to the downregulated metabolic pathways in PS cDKO mice, such as TCA cycle, glyoxylate and dicarboxylate metabolism; arginine biosynthesis; d-glutamine and d-glutamate metabolism; glutathione metabolism; glyoxylate and dicarboxylate metabolism; and glutathione metabolism, etc. In addition, *Mucispirillum* is positively related to xylitol and galactonic acid, and the former is related to the pathway of pentose and glucuronate interconversions, which was reported in pathogenesis of glycometabolism disease [59]. Overall, as a kind of typical AD rodent model, PS cDKO mice in both 2-month age and 6-month age showed metabolomic and microbiotic changes (Fig. 8).

As mentioned above, the correlation analysis of the gut microbiota in genus level and the differential urinary metabolites showed that in PS cDKO and WT mice, metabolites related to norank*_f_Muribaculaceae*, *Faecalibaculum* and *Odoribacter* might be closely associated with the host energy metabolism and amino acid metabolism. In contrast, metabolites related to *Lactobacillus*, *Lachnospiraceae_NK4A136_group*, and *Mucispirillum* may be mainly associated with the host TCA circle and glycine metabolism. All of evidences displayed that there were remarkable crosstalks between gut microbes and internal metabolites.

## Supplementary Information

**Additional file 1: Table S1.** Differential metabolites between PS cDKO group and Control group at age of 2 months and 6 months.

## Data Availability

The data are included in the article as figures and its additional files.

## References

[1] Adlimoghaddam A, Snow WM, Stortz G, Perez C, Djordjevic J, Goertzen AL (2019). Regional hypometabolism in the 3xTg mouse model of Alzheimer's disease. Neurobiol Dis.

[2] Antuono PG, Jones JL, Wang Y, Li SJ (2001). Decreased glutamate + glutamine in Alzheimer's disease detected in vivo with (1)H-MRS at 0.5 T. Neurology.

[3] Blass JP, Sheu RK, Gibson GE (2000). Inherent abnormalities in energy metabolism in Alzheimer disease. Interaction with cerebrovascular compromise. Ann N Y Acad Sci.

[4] Bonds JA, Kuttner-Hirshler Y, Bartolotti N, Tobin MK, Pizzi M, Marr R (2015). Presenilin-1 dependent neurogenesis regulates hippocampal learning and memory. PLoS ONE.

[5] Castro-Gomez S, Binder J, Heneka MT (2019). Neuroinflammation as motor of Alzheimer's disease. Nervenarzt.

[6] Cattaneo A, Cattane N, Galluzzi S, Provasi S, Lopizzo N, Festari C (2017). Association of brain amyloidosis with pro-inflammatory gut bacterial taxa and peripheral inflammation markers in cognitively impaired elderly. Neurobiol Aging.

[7] Chang DH, Rhee MS, Ahn S, Bang BH, Oh JE, Lee HK (2015). *Faecalibaculum rodentium* gen. nov., sp. nov., isolated from the faeces of a laboratory mouse. Antonie Van Leeuwenhoek.

[8] Chen Q, Nakajima A, Choi SH, Xiong X, Tang YP (2008). Loss of presenilin function causes Alzheimer's disease-like neurodegeneration in the mouse. J Neurosci Res.

[9] Chen ZC, Zhong CJ (2013). Decoding Alzheimer's disease from perturbed cerebral glucose metabolism: Implications for diagnostic and therapeutic strategies. Prog Neurobiol.

[10] Cheng D, Chang H, Ma S, Guo J, She G, Zhang F (2018). Tiansi liquid modulates gut microbiota composition and tryptophan(-)kynurenine metabolism in rats with hydrocortisone-induced depression. Molecules.

[11] Chogtu B, Arivazhahan A, Kunder SK, Tilak A, Sori R, Tripathy A (2018). Evaluation of acute and chronic effects of d-galactose on memory and learning in Wistar rats. Clin Psychopharmacol Neurosci.

[12] Cox LM, Weiner HL (2018). Microbiota signaling pathways that influence neurologic disease. Neurotherapeutics.

[13] Cui GH, Wu J, Mou FF, Xie WH, Wang FB, Wang QL (2018). Exosomes derived from hypoxia-preconditioned mesenchymal stromal cells ameliorate cognitive decline by rescuing synaptic dysfunction and regulating inflammatory responses in APP/PS1 mice. FASEB J.

[14] De Vadder F, Kovatcheva-Datchary P, Goncalves D, Vinera J, Zitoun C, Duchampt A (2014). Microbiota-generated metabolites promote metabolic benefits via gut-brain neural circuits. Cell.

[15] Dhaliwal J, Kannangara TS, Vaculik M, Xue Y, Kumar KL, Maione A (2018). Adult hippocampal neurogenesis occurs in the absence of Presenilin 1 and Presenilin 2. Sci Rep.

[16] Dong Z, Yan L, Huang G, Zhang L, Mei B, Meng B (2014). Ibuprofen partially attenuates neurodegenerative symptoms in presenilin conditional double-knockout mice. Neuroscience.

[17] Edgar RC (2013). UPARSE: highly accurate OTU sequences from microbial amplicon reads. Nat Methods.

[18] Fadrosh DW, Ma B, Gajer P, Sengamalay N, Ott S, Brotman RM (2014). An improved dual-indexing approach for multiplexed 16S rRNA gene sequencing on the Illumina MiSeq platform. Microbiome.

[19] Giau VV, Wu SY, Jamerlan A, An SSA, Kim SY, Hulme J (2018). Gut microbiota and their neuroinflammatory implications in Alzheimer's disease. Nutrients.

[20] Gonzalez-Dominguez R, Garcia-Barrera T, Vitorica J, Gomez-Ariza JL (2014). Region-specific metabolic alterations in the brain of the APP/PS1 transgenic mice of Alzheimer's disease. Biochim Biophys Acta.

[21] Harada K, Nakato K, Yarimizu J, Yamazaki M, Morita M, Takahashi S (2012). A novel glycine transporter-1 (GlyT1) inhibitor, ASP2535 (4-[3-isopropyl-5-(6-phenyl-3-pyridyl)-4H-1,2,4-triazol-4-yl]-2,1,3-benzoxadiazol e), improves cognition in animal models of cognitive impairment in schizophrenia and Alzheimer's disease. Eur J Pharmacol.

[22] Hardham JM, King KW, Dreier K, Wong J, Strietzel C, Eversole RR (2008). Transfer *of Bacteroides splanchnicus* to *Odoribacter* gen. nov. as *Odoribacter splanchnicus* comb. Nov., and description of *Odoribacter denticanis* sp. nov., isolated from the crevicular spaces of canine periodontitis patients. Int J Syst Evol Microbiol.

[23] Herp S, Brugiroux S, Garzetti D, Ring D, Jochum LM, Beutler M (2019). *Mucispirillum schaedleri* antagonizes salmonella virulence to protect mice against colitis. Cell Host Microbe.

[24] Ho L, Ono K, Tsuji M, Mazzola P, Singh R, Pasinetti GM (2018). Protective roles of intestinal microbiota derived short chain fatty acids in Alzheimer's disease-type beta-amyloid neuropathological mechanisms. Expert Rev Neurother.

[25] Hogan-Cann AD, Anderson CM (2016). Physiological Roles of Non-Neuronal NMDA Receptors. Trends Pharmacol Sci.

[26] Horie M, Miura T, Hirakata S, Hosoyama A, Sugino S, Umeno A (2017). Comparative analysis of the intestinal flora in type 2 diabetes and nondiabetic mice. Exp Anim.

[27] Huo Z, Yu L, Yang J, Zhu Y, Bennett DA, Zhao J (2020). Brain and blood metabolome for Alzheimer's dementia: findings from a targeted metabolomics analysis. Neurobiol Aging.

[28] Jia J, Wei C, Chen S, Li F, Tang Y, Qin W (2018). The cost of Alzheimer's disease in China and re-estimation of costs worldwide. Alzheimers Dement.

[29] Jiang X, Zhang D, Shi J, Chen Y, Zhang P, Mei B (2009). Increased Inflammatory Response Both in Brain and in Periphery in Presenilin 1 and Presenilin 2 Conditional Double Knock-Out Mice. J Alzheimers Dis.

[30] Kaddurah-Daouk R, Rozen S, Matson W, Han X, Hulette CM, Burke JR (2011). Metabolomic changes in autopsy-confirmed Alzheimer's disease. Alzheimers Dement.

[31] Kao YC, Ho PC, Tu YK, Jou IM, Tsai KJ (2020). Lipids and Alzheimer's Disease. Int J Mol Sci.

[32] Kim MS, Kim Y, Choi H, Kim W, Park S, Lee D (2020). Transfer of a healthy microbiota reduces amyloid and tau pathology in an Alzheimer's disease animal model. Gut.

[33] Knezovic A, Barilar JO, Babic A, Bagaric R, Farkas V, Riederer P (2018). Glucagon-like peptide-1 mediates effects of oral galactose in streptozotocin-induced rat model of sporadic Alzheimer's disease. Neuropharmacology.

[34] Koh A, De Vadder F, Kovatcheva-Datchary P, Backhed F (2016). From dietary fiber to host physiology: short-chain fatty acids as key bacterial metabolites. Cell.

[35] Kunkle BW, Grenier-Boley B, Sims R, Bis JC, Damotte V, Naj AC (2019). Genetic meta-analysis of diagnosed Alzheimer's disease identifies new risk loci and implicates Abeta, tau, immunity and lipid processing. Nat Genet.

[36] Kurbatova N, Garg M, Whiley L, Chekmeneva E, Jimenez B, Gomez-Romero M (2020). Urinary metabolic phenotyping for Alzheimer's disease. Sci Rep.

[37] Lagkouvardos I, Lesker TR, Hitch TCA, Galvez EJC, Smit N, Neuhaus K (2019). Sequence and cultivation study of Muribaculaceae reveals novel species, host preference, and functional potential of this yet undescribed family. Microbiome.

[38] Lee D, Aoki C (2012). Presenilin conditional double knockout mice exhibit decreases in drebrin a at hippocampal CA1 synapses. Synapse.

[39] Li W, Yu J, Liu Y, Huang X, Abumaria N, Zhu Y (2014). Elevation of brain magnesium prevents synaptic loss and reverses cognitive deficits in Alzheimer's disease mouse model. Mol Brain.

[40] Li Y, Luan Y, Yue X, Xiang F, Mao D, Cao Y (2019). Effects of Codonopis bulleynana forest ex diels on Deferribacteres in constipation predominant intestine tumor: Differential analysis. Saudi J Biol Sci.

[41] Lim S, Chang DH, Ahn S, Kim BC (2016). Whole genome sequencing of "Faecalibaculum rodentium" ALO17, isolated from C57BL/6J laboratory mouse feces. Gut Pathogens.

[42] Liu GS, Weinger JG, Lu ZL, Xue F, Sadeghpour S (2016). Efficacy and Safety of MMFS-01, a synapse density enhancer, for treating cognitive impairment in older adults: a randomized, double-blind. placebo-controlled trial. J Alzheimers Dis.

[43] Liu YY, Wei MY, Yue KX, Hu MX, Li SZ, Men LH (2018). Study on urine metabolic profile of A beta 25–35-induced Alzheimer's disease using UHPLC-Q-TOF-MS. Neuroscience.

[44] Ma W, Song J, Wang H, Shi F, Zhou N, Jiang J (2019). Chronic paradoxical sleep deprivation-induced depression-like behavior, energy metabolism and microbial changes in rats. Life Sci.

[45] Ma WN, Zhou MM, Gou XJ, Zhao L, Cen F, Xu Y (2018). Urinary metabolomic study of chlorogenic acid in a rat model of chronic sleep deprivation using gas chromatography-mass spectrometry. Int J Genomics.

[46] Maeda J, Nishida M, Takikawa H, Yoshida H, Azuma T, Yoshida M (2010). Inhibitory effects of sulfobacin B on DNA polymerase and inflammation. Int J Mol Med.

[47] Megur AA-O, Baltriukienė DA-O, Bukelskienė V, Burokas AA-O (2020). The microbiota-gut-brain axis and Alzheimer's disease: neuroinflammation is to blame?. Nutrients.

[48] Morris RG (2013). NMDA receptors and memory encoding. Neuropharmacology.

[49] Nagai F, Morotomi M, Watanabe Y, Sakon H, Tanaka R (2010). *Alistipes indistinctus* sp. nov. and *Odoribacter laneus* sp. nov., common members of the human intestinal microbiota isolated from faeces. Int J Syst Evol Microbiol.

[50] Olson BL, Holshouser BA, Britt W, Mueller C, Baqai W, Patra S (2008). Longitudinal metabolic and cognitive changes in mild cognitive impairment patients. Alzheimer Dis Assoc Disord.

[51] Ormerod KL, Wood DL, Lachner N, Gellatly SL, Daly JN, Parsons JD (2016). Genomic characterization of the uncultured Bacteroidales family S24–7 inhabiting the guts of homeothermic animals. Microbiome.

[52] Patel AB, de Graaf RA, Mason GF, Rothman DL, Shulman RG, Behar KL (2005). The contribution of GABA to glutamate/glutamine cycling and energy metabolism in the rat cortex in vivo. Proc Natl Acad Sci USA.

[53] Braidy N, Zarka M, Welch J, Bridge W (2015). Therapeutic approaches to modulating glutathione levels as a pharmacological strategy in Alzheimer's disease. Curr Alzheimer Res.

[54] Pichler M, Coskun OK, Ortega-Arbulu AS, Conci N, Worheide G, Vargas S (2018). A 16S rRNA gene sequencing and analysis protocol for the Illumina MiniSeq platform. Microbiologyopen.

[55] Fing R, Wang H (2004). Forebrain degeneration and ventricle enlargement caused by double knockout of Alzheimer's presenilin-1 and presenilin-2. Neuron.

[56] Rutsch A, Kantsjo JB, Ronchi F (2020). The Gut-Brain Axis: how microbiota and host inflammasome influence brain physiology and pathology. Front Immunol.

[57] Salek RM, Xia J, Innes A, Sweatman BC, Adalbert R, Randle S (2010). A metabolomic study of the CRND8 transgenic mouse model of Alzheimer's disease. Neurochem Int.

[58] Salkovic-Petrisic M, Osmanovic-Barilar J, Knezovic A, Hoyer S, Mosetter K, Reutter W (2014). Long-term oral galactose treatment prevents cognitive deficits in male Wistar rats treated intracerebroventricularly with streptozotocin. Neuropharmacology.

[59] Sandholm N, Van Zuydam N, Ahlqvist E, Juliusdottir T, Deshmukh HA, Rayner NW (2017). The genetic landscape of renal complications in type 1 diabetes. J Am Soc Nephrol.

[60] Saura CA, Choi SY, Beglopoulos V, Malkani S, Zhang D, Shankaranarayana Rao BS (2004). Loss of presenilin function causes impairments of memory and synaptic plasticity followed by age-dependent neurodegeneration. Neuron.

[61] Shenghua P, Ziqin Z, Shuyu T, Huixia Z, Xianglu R, Jiao G (2020). An integrated fecal microbiome and metabolome in the aged mice reveal anti-aging effects from the intestines and biochemical mechanism of FuFang zhenshu TiaoZhi(FTZ). Biomed Pharmacother.

[62] Sibai M, Altuntas E, Yildirim B, Ozturk G, Yildirim S, Demircan T (2020). Microbiome and longevity: high abundance of longevity-linked muribaculaceae in the gut of the long-living rodentspalax leucodon. Omics.

[63] Silva YP, Bernardi A, Frozza RL (2020). The role of short-chain fatty acids from gut microbiota in gut-brain communication. Front Endocrinol (Lausanne).

[64] Slutsky I, Abumaria N, Wu LJ, Huang C, Zhang L, Li B (2010). Enhancement of learning and memory by elevating brain magnesium. Neuron.

[65] Smith BJ, Miller RA, Ericsson AC, Harrison DC, Strong R, Schmidt TM (2019). Changes in the gut microbiome and fermentation products concurrent with enhanced longevity in acarbose-treated mice. BMC Microbiol.

[66] Song J, Ma W, Gu X, Zhao L, Jiang J, Xu Y (2019). Metabolomic signatures and microbial community profiling of depressive rat model induced by adrenocorticotrophic hormone. J Transl Med.

[67] Su J, Gu J, Dong Z, Mei B (2013). Ibuprofen rescues abnormalities in periodontal tissues in conditional presenilin 1 and presenilin 2 double knockout mice. Int J Mol Sci.

[68] Sun BL, Li WW, Wang J, Xu YL, Sun HL, Tian DY (2019). Gut microbiota alteration and its time course in a tauopathy mouse model. J Alzheimers Dis.

[69] Sun QF, Weinger JG, Mao F, Liu GS (2016). Regulation of structural and functional synapse density by L-threonate through modulation of intraneuronal magnesium concentration. Neuropharmacology.

[70] Tian X, Yu Z, Feng P, Ye Z, Li R, Liu J (2019). Lactobacillus plantarum TW1-1 alleviates diethylhexylphthalate-induced testicular damage in mice by modulating gut microbiota and decreasing inflammation. Front Cell Infect Microbiol.

[71] Vogt NM, Kerby RL, Dill-McFarland KA, Harding SJ, Merluzzi AP, Johnson SC (2017). Gut microbiome alterations in Alzheimer's disease. Sci Rep.

[72] Volk JK, Nyström EEL, van der Post S, Abad BM, Schroeder BA-O, Johansson Å (2019). The Nlrp6 inflammasome is not required for baseline colonic inner mucus layer formation or function. J Exp Med.

[73] Walker A, Pfitzner B, Harir M, Schaubeck M, Calasan J, Heinzmann SS (2017). Sulfonolipids as novel metabolite markers of Alistipes and Odoribacter affected by high-fat diets. Sci Rep.

[74] Wang Q, Garrity GM, Tiedje JM, Cole JR (2007). Naive Bayesian classifier for rapid assignment of rRNA sequences into the new bacterial taxonomy. Appl Environ Microbiol.

[75] Wang X, Sun G, Feng T, Zhang J, Huang X, Wang T (2019). Sodium oligomannate therapeutically remodels gut microbiota and suppresses gut bacterial amino acids-shaped neuroinflammation to inhibit Alzheimer's disease progression. Cell Res.

[76] Wei MY, Liu ZY, Liu YY, Li SZ, Hu MX, Yue KX (2019). Urinary and plasmatic metabolomics strategy to explore the holistic mechanism of lignans in *S. chinensis* in treating Alzheimer's disease using UPLC-Q-TOF-MS. Food Function.

[77] Wilkins JM, Trushina E (2017). Application of metabolomics in Alzheimer's disease. Front Neurol.

[78] Wines-Samuelson M, Schulte EC, Smith MJ, Aoki C, Liu X, Kelleher RJ (2010). Characterization of age-dependent and progressive cortical neuronal degeneration in presenilin conditional mutant mice. PLoS ONE.

[79] World Health Organization. Dementia fact sheets (2019). https://www.who.int/news-room/fact-sheets/detail/dementia/. Accessed 14 May 2019.

[80] Wu G, Fang YZ, Yang S, Lupton JR, Turner ND (2004). Glutathione metabolism and its implications for health. J Nutr.

[81] Yan L, Li L, Han W, Pan B, Xue X, Mei B (2013). Age-related neuropsychiatric symptoms in presenilins conditional double knockout mice. Brain Res Bull.

[82] Ye BS, Lee Y, Kwak K, Park Y-H, Ham JH, Lee JJ (2016). Posterior ventricular enlargement to differentiate dementia with Lewy bodies from Alzheimer’s disease. J Alzheimers Dis.

[83] Yilmaz A, Ugur Z, Bisgin H, Akyol S, Bahado-Singh R, Wilson G (2020). Targeted metabolic profiling of urine highlights a potential biomarker panel for the diagnosis of Alzheimer's disease and mild cognitive impairment: a pilot study. Metabolites.

[84] Zagato E, Pozzi C, Bertocchi A, Schioppa T, Saccheri F, Guglietta S (2020). Endogenous murine microbiota member *Faecalibaculum rodentium* and its human homologue protect from intestinal tumour growth. Nat Microbiol.

[85] Zetterberg H, Burnham SC (2019). Blood-based molecular biomarkers for Alzheimer's disease. Mol Brain.

[86] Zhang L, Wang Y, Xiayu X, Shi C, Chen W, Song N (2017). Altered gut microbiota in a mouse model of Alzheimer's disease. J Alzheimers Dis.

[87] Zhang X, Zhang N, Kan J, Sun R, Tang S, Wang Z (2020). Anti-inflammatory activity of alkali-soluble polysaccharides from *Arctium lappa* L. and its effect on gut microbiota of mice with inflammation. Int J Biol Macromol.

[88] Zhang Z, Cao H, Song N, Zhang L, Cao Y, Tai J (2020). Long-term hexavalent chromium exposure facilitates colorectal cancer in mice associated with changes in gut microbiota composition. Food Chem Toxicol.

[89] Zhao Y, Deng H, Li K, Wang L, Wu Y, Dong X (2019). Trans-cinnamaldehyde improves neuroinflammation-mediated NMDA receptor dysfunction and memory deficits through blocking NF-kappaB pathway in presenilin1/2 conditional double knockout mice. Brain Behav Immun.

[90] Zhou ZL, Jia XB, Sun MF, Zhu YL, Qiao CM, Zhang BP (2019). Neuroprotection of fasting mimicking diet on MPTP-induced Parkinson's disease mice via gut microbiota and metabolites. Neurotherapeutics.

[91] Zhu S, Jiang Y, Xu K, Cui M, Ye W, Zhao G (2020). The progress of gut microbiome research related to brain disorders. J Neuroinflammation.

[92] Zhuang ZQ, Shen LL, Li WW, Fu X, Zeng F, Gui L (2018). Gut microbiota is altered in patients with alzheimer's disease. J Alzheimers Dis.

