# Supplementary material for: Urinary metabolomic changes and microbiotic alterations in presenilin1/2 conditional double knockout mice
Source: J Transl Med. 2021 Aug 16;19:351. doi: 10.1186/s12967-021-03032-9 (PMC8365912; doi:10.1186/s12967-021-03032-9)
Supplement: Supplementary file 1 — Additional file 1: Table S1. Differential metabolites between PS cDKO group and Control group at age of 2 months and 6 months. [file 12967_2021_3032_MOESM1_ESM.docx]

**Additional file 1: Table S1. Differential metabolites between PS cDKO group and Control group at age of 2 months and 6 months.**

| **Clusters** | **Metabolites** | **RT**  **( min)** | **Mz** | **Fold Change^a^**  **(cDKO *vs* WT)** | | **p value**  **(cDKO *vs* WT)** | | **VIP value^b^**  **(cDKO *vs* WT)** | | **Metabolic Pathways** | **Trend** | |
| --- | --- | --- | --- | --- | --- | --- | --- | --- | --- | --- | --- | --- |
|  |  |  |  | **2 Mon** | **6 Mon** | **2 Mon** | **6 Mon** | **2 Mon** | **6 Mon** |  | **2 Mon** | **6 Mon** |
| **Ⅰ** | **Butyric acid** | 10.69 | 88 | 3.22 | 1.76 | 0.009 | 0.494 | 1.37 | 0.27 | Others | **↑** | **↑** |
|  | **Glycerate** | 10.74 | 106 | 7.54 | 0.69 | 0.004 | 0.261 | 1.16 | 0.19 | Glyoxylate and dicarboxylate metabolism | **↑*** | **↓** |
|  | **Glycolic acid** | 5.51 | 76 | 0.45 | 1.15 | 0.008 | 0.333 | 4.11 | 0.23 | Others | **↓** | **↑** |
|  | **Erythritol** | 14.88 | 122 | 0.78 | 1.96 | 0.002 | 0.026 | 2.16 | 0.47 | Others | **↓** | **↑** |
|  | **D-Galactose** | 23.54 | 489 | 0.72 | 0.95 | 0.005 | 0.497 | 1.36 | 0.19 | Others | **↓** | **↓** |
|  | **D-Glucose** | 23.44 | 180 | 0.52 | 0.91 | 0.001 | 0.299 | 1.70 | 0.57 | Starch and sucrose metabolism | **↓*** | **↓** |
| **Ⅱ** | **Xylitol** | 23.41 | 152 | 0.60 | 0.80 | 0.031 | 0.001 | 2.94 | 1.23 | Pentose and glucuronate interconversions | **↓*** | **↓*** |
|  | **Glycine** | 9.25 | 75 | 0.29 | 0.24 | 0.013 | 0.000 | 1.63 | 1.24 | Glycine, serine and threonine metabolism;glyoxylateanddicarboxylate metabolism; glutathione metabolism | **↓*** | **↓*** |
| **Ⅲ** | **Succinic acid** | 11.13 | 118 | 0.56 | 0.47 | 0.001 | 0.007 | 0.35 | 1.75 | Citrate cycle (TCA cycle); alanine, aspartate and glutamate metabolism | **↓** | **↓*** |
|  | **Threonic acid** | 16.94 | 136 | 0.73 | 0.56 | 0.004 | 0.029 | 0.31 | 1.33 | Others | **↓** | **↓** |
|  | **5-Hydroxyindole-3- Acetate** | 19.38 | 290 | 0.70 | 0.88 | 0.201 | 0.001 | 0.47 | 1.57 | Others | **↓** | **↓** |
|  | **Glutamate** | 17.36 | 147 | 0.78 | 0.29 | 0.055 | 0.000 | 0.35 | 1.06 | Glyoxylate and dicarboxylate metabolism; arginine biosynthesis; D-glutamine and D-glutamate metabolism; glutathione metabolism;alanine, aspartate and glutamate metabolism | **↓** | **↓*** |
|  | **Galactonic acid** | 26.28 | 172 | 0.86 | 0.34 | 0.090 | 0.000 | 0.22 | 1.21 | Others | **↓** | **↓** |
|  | ***cis*-Aconitate** | 21.26 | 176 | 0.70 | 0.52 | 0.195 | 0.000 | 0.65 | 1.88 | Citrate cycle (TCA cycle); glyoxylate and dicarboxylate metabolism | **↓** | **↓*** |
|  | **Citric acid** | 22.71 | 192 | 0.70 | 0.20 | 0.335 | 0.000 | 0.10 | 5.28 | Citrate cycle (TCA cycle); alanine, aspartate and glutamate metabolism | **↓** | **↓*** |
|  | **Isovalerylglycine** | 15.32 | 159 | 1.75 | 2.18 | 0.201 | 0.000 | 0.01 | 2.18 | Others | **↑** | **↑** |
|  | ***m*-Cresol** | 7.51 | 108 | 1.30 | 3.79 | 0.193 | 0.003 | 0.22 | 3.79 | Others | **↑** | **↑** |

**a Fold change was calculated as the ratio of the average relative level between the two groups (FC value = PS cDKO/WT). b VIP was obtained from OPLS-DA.**

*** Metabolites involved in pathway analysis (impact factor ≥ 0.1). ↑ : Metabolites increased in PS cDKO group; ↓: Metabolites decreased in PS cDKO group.**
